# A comparison of modelling techniques for computing wall stress in abdominal aortic aneurysms

**DOI:** 10.1186/1475-925X-6-38

**Published:** 2007-10-19

**Authors:** Barry J Doyle, Anthony Callanan, Timothy M McGloughlin

**Affiliations:** 1 Centre for Applied Biomedical Engineering Research (CABER), Department of Mechanical and Aeronautical Engineering and Materials and Surface Science Institute, University of Limerick, Ireland

## Abstract

**Background:**

Aneurysms, in particular abdominal aortic aneurysms (AAA), form a significant portion of cardiovascular related deaths. There is much debate as to the most suitable tool for rupture prediction and interventional surgery of AAAs, and currently maximum diameter is used clinically as the determining factor for surgical intervention. Stress analysis techniques, such as finite element analysis (FEA) to compute the wall stress in patient-specific AAAs, have been regarded by some authors to be more clinically important than the use of a "one-size-fits-all" maximum diameter criterion, since some small AAAs have been shown to have higher wall stress than larger AAAs and have been known to rupture.

**Methods:**

A patient-specific AAA was selected from our AAA database and 3D reconstruction was performed. The AAA was then modelled in this study using three different approaches, namely, *AAA*(*SIMP*), *AAA*(*MOD*) and *AAA*(*COMP*), with each model examined using linear and non-linear material properties. All models were analysed using the finite element method for wall stress distributions.

**Results:**

Wall stress results show marked differences in peak wall stress results between the three methods. Peak wall stress was shown to reduce when more realistic parameters were utilised. It was also noted that wall stress was shown to reduce by 59% when modelled using the most accurate non-linear complex approach, compared to the same model without intraluminal thrombus.

**Conclusion:**

The results here show that using more realistic parameters affect resulting wall stress. The use of simplified computational modelling methods can lead to inaccurate stress distributions. Care should be taken when examining stress results found using simplified techniques, in particular, if the wall stress results are to have clinical importance.

## Background

Cardiovascular disease is the leading cause of morbidity and premature deaths of modern era medicine and aneurysms are a major contributor to the poor clinical outcomes. Aneurysm, meaning widening, can be defined as a permanent and irreversible localised dilatation of a vessel [1]. Although this disease can form in any blood vessel, artery or vein, the more serious aneurysms occur in the abdominal aorta, the brain arteries, and the heart and thoracic aorta. The vast majority of these aneurysms occur in the abdominal aorta, and are termed Abdominal Aortic Aneurysm (AAA). There is much debate amongst researchers and surgeons as to the most appropriate criteria for surgical intervention of unruptured AAAs [2-6]. Currently, the maximum diameter of the aneurysm is the most commonly used determinant of surgical intervention, with clinicians opting for surgery when the AAA exceeds 5 cm in transverse diameter [4,5,7]. Although this maximum diameter criterion can be justified, as the rupture risk for an AAA is clearly related to its maximal diameter [5,8], it is also known that small AAAs can rupture [5,9-11]. Nicholls et al. [11] reported in one study that 10% to 24% of ruptured aneurysms were 5 cm or less in diameter, and therefore there is clearly a need for an improved predictor of rupture.

It is believed by many researchers that stress analyses are more accurate indicators than diameter for predicting rupture [2,5-7,12-14], and it is clear that a reliable method of predicting AAA rupture has definite clinical importance [2,3,6,7,13].

It has been previously documented how AAA formation is accompanied by an increase in wall stress [2,7], and a decrease in wall strength [15-17]. It has been identified that aneurysm wall stress does not follow the traditional Law of Laplace, in that AAAs with equivalent diameters and pressures could have largely different stress distributions [2,3,7,13]. Researchers have been using stress analysis techniques, such as finite element analysis (FEA), to compute stresses within AAAs for some time now [2,5,7,13], but this work has yet to be validated experimentally using realistic AAA models. Morris et al. [18] showed experimentally the stress contours in an idealised AAA using the photoelastic method, which was later validated numerically by Callanan et al. [19]. Previous numerical studies have shown how asymmetry and diameter affect wall stress [2], and also how this wall stress is distributed within a realistic AAA [5,7,13,20]. Dobrin [21] suggested that the presence of ILT neither reduces the luminal pressure exerted on the wall nor offers a retractive force and thus has no effect on the wall stress. This ILT was later reported to significantly reduce the stress acting on the AAA wall [13] and to act as a mechanical cushion [13,17], although some researchers dispute this [22].

The purpose of this study is to analyse and compare the differences associated with finite element analysis (FEA) modelling of AAAs. Many researchers have used simplified AAA models in their studies [2,23-25], compared to others who have used much more complex, realistic methods [2,3,7,13,20,26-28]. The more common simplifying assumptions include treating the biomechanical material properties of the vessel as linearly elastic and excluding the intraluminal thrombus (ILT) from the model. One patient-specific AAA was examined in this study. The model was examined from various aspects of complexity, starting with a simplified approach, *AAA*(*SIMP*), followed by a moderate method, *AAA*(*MOD*), and then a complex technique, *AAA*(*COMP*), with results and differences contrasted and compared. In this study, simplified refers to the use of common simplifying assumptions. Moderate refers to the use of a limited amount of simplifying assumptions, with complex inferring the inclusion of ILT. All models were examined using both linear and non-linear material properties.

## Methods

A patient suffering from an AAA was selected from our AAA database which included modest levels of intraluminal thrombus (ILT). The study subject was male, and the AAA maximum transverse diameter was 5.1 cm, and so had exceeded the 5 cm threshold for surgical intervention. The total length of the AAA was 13.2 cm, had a total volume of 176.7 cm^3^, of which 67.5 cm^3 ^was ILT. For clinically meaningful stress analysis of AAAs, patient-specific computed tomography (CT) scans were obtained for the patient. This CT data was then reconstructed using commercially available software (Mimics v10.0, Materialise, Belgium). In total, six models were created and analysed. Three different methods of reconstruction were utilised to create the AAA, with each model examined using both linear and non-linear material properties. For the purpose of this study, the AAAs modelled using the first set of simplifying assumptions will be referred to as *AAA*(*SIMP*)_*L *_and *AAA*(*SIMP*)_*NL*_, with the subscripts *L *and *NL *referring to linear and non-linear material properties, respectively. The second set of AAAs will be referred to as *AAA*(*MOD*)_*L *_and *AAA*(*MOD*)_*NL*_, due to the use of moderate simplifying assumptions. The third set of AAAs, which used a more complex method of analysis will be referred to as *AAA*(*COMP*)_*L *_and *AAA*(*COMP*)_*NL*_. All stress analyses were performed using the finite element analysis software ABAQUS v6.6-2 (Dassault Systemes, SIMULIA, Rhode Island, USA) on a standard desktop with a 3.2 GHz processor and 4GB of RAM.

### 3D reconstruction

**Figure 1:**
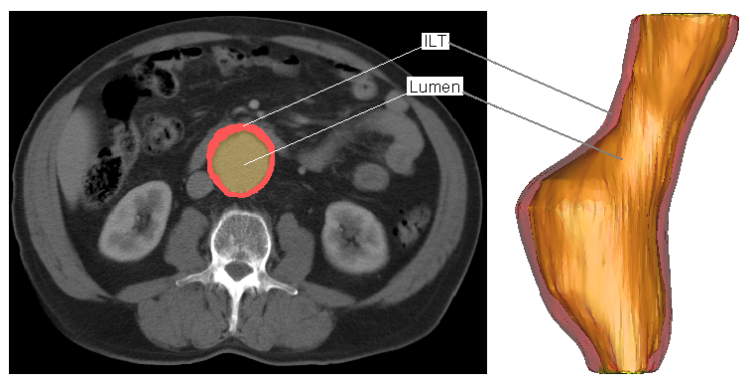
**Typical CT scan and 3D reconstruction of patient suffering from an AAA**. On the left shows a typical CT scan after segmentation using Mimics v10.0, with the ILT (red) and lumen (yellow) clearly distinguishable. 3D reconstruction of examined AAA is shown on right.

Spiral CT data was then used to reconstruct the infrarenal section of the aorta. As CT scanning is routinely performed on AAA patients scheduled for repair, collection of this information involved no extra participation by the study subject. Digital files in Digital Imaging and Communications in Medicine (DICOM) file format, containing cross-sectional information was then imported to Mimics for reconstruction. A Hounsfield unit (HU) thresholding technique was then applied to each CT slice in order to identify the region of interest. A HU value of 226 is sufficient to identify the lumen region of the AAA due to the non-ionic contrast. This contrast dye is an organic solution that makes internal bodily structures visible. The ILT regions of the AAA must be assigned a lower HU value, as the material has a pixel intensity that is closer to fat than bone. A HU value of 0 is sufficient to distinguish the ILT from the surrounding tissue. For all models reconstructed, the iliac arteries have been omitted as with previous research [5]. Figure 1 shows the conversion from CT scan to 3D model. On the left the thresholding and segmentation process highlights both the ILT and lumen of the AAA, with the software generating 3D reconstructions, shown on the right. For the *AAA*(*SIMP*) and *AAA*(*MOD*) models, the ILT was omitted, and so the reconstruction consisted of a single layer wall of uniform thickness.

#### AAA(SIMP)

For this set of AAAs, the ILT was omitted from the reconstruction, and was instead incorporated into the full AAA model as lumen. Previous work [5,6] has also taken this approach to AAA modelling. The thickness of the aortic wall is not easily identifiable from CT scans. Previous research [2,5-7,18,19,25,26,29] has used wall thickness varying from 1–2 mm. As no information was available from the CT scans about the wall thickness of each AAA, in this study the wall was assumed to be of uniform thickness throughout the model and was set to 1.5 mm [2,26,29].

#### AAA(MOD)

When reconstructing the *AAA*(*MOD*) models, the surface polylines created using Mimics v10.0, were exported to ProEngineer Wildfire 3.0 (Parametric Technology Corporation, Needham, M.A., USA) in order to create a 3D wall. The original polylines were offset by 1.5 mm [2,26,29] so as to create a second artificial surface offset from the original surface. These surfaces were then connected so as to form a realistic uniform 3D aortic wall.

#### AAA(COMP)

For this AAA, the ILT region was included into the models. Therefore, the ILT is a completely different entity to that of the AAA wall, with the two sections tied together using realistic constraints. This is a more realistic representation of the actual AAA with the ILT. An artificial wall was created to represent the AAA wall and, like earlier, was set to be a uniform 1.5 mm in thickness [2,26,29].

#### Model smoothing

**Figure 2:**
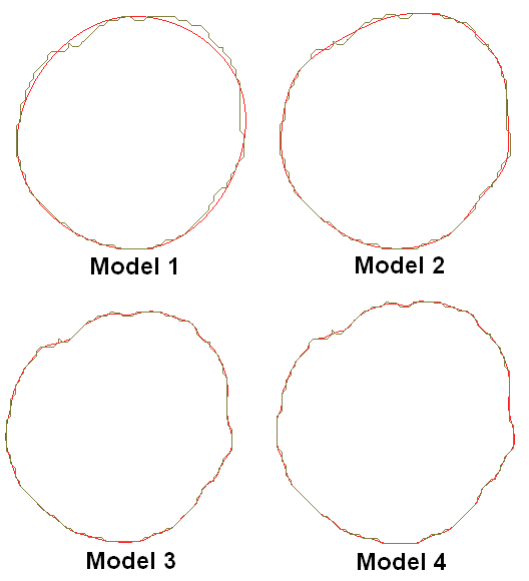
**Effect of axial smoothing on the polylines constructed from the CT scans**. Model 1 consists of 7 control points, Model 2 has 20 control points, Model 3 has 50 control points, and Model 4 has 70 control points per polylines slice. Green line is the original polyline from the CT scan slice, with the red line being the new smoothed polyline.

**Figure 3:**
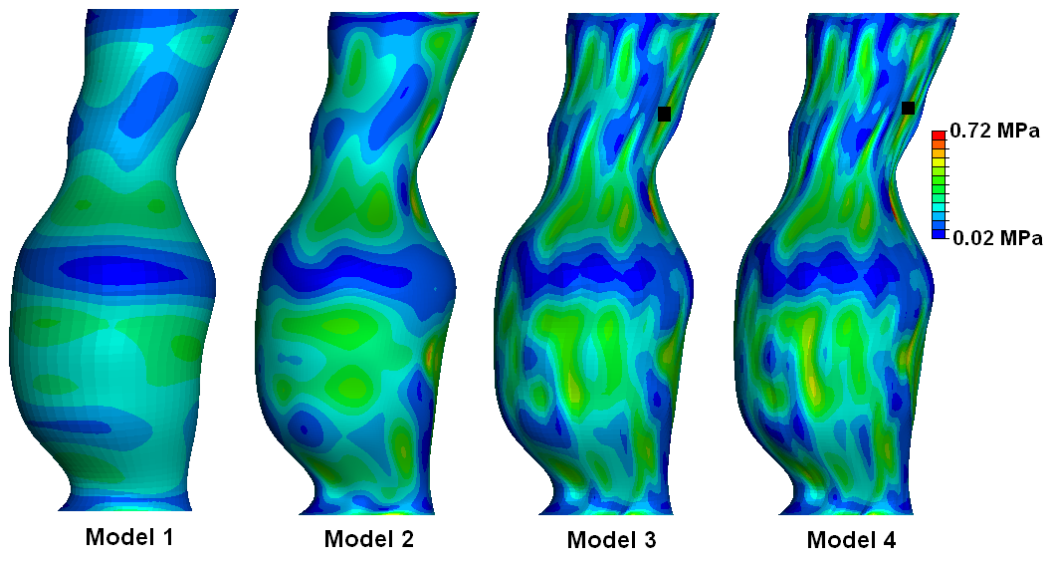
**Resulting wall stress distributions of the various degrees of smoothing examined**. Wall stress results are normalised to the peak stress found in Model 4. Black mark indicates abnormal locations of elevated wall stress in Models 3 and 4.

**Figure 4:**
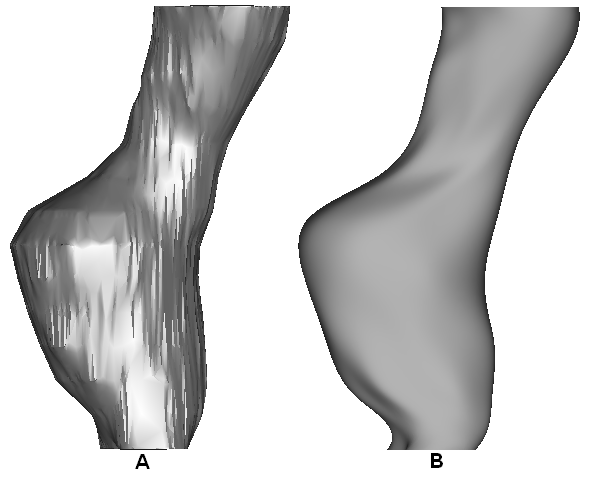
**Effect of smoothing on the reconstructed AAA**. The optimum smoothing factor removes unwanted surface detail without over-smoothing the AAA. (A) Shows the rough model without smoothing and (B) shows the effect of a 20 control point axial smoothing factor. Model shown in the lateral view.

Each of these 3D models were initially quite rough containing sharp edges and surface artefacts, and required smoothing to ensure that the model could be readily meshed and that stress analyses could be performed. The method of reconstruction and smoothing was validated from previous work by our group, [30,31] and it was shown that there are negligible differences between the two methods. In order to determine the optimum level of smoothing for these reconstructions, four degrees of smoothing were examined. These four smoothing levels were based on axial smoothing of individual polyline slices created from the CT scans. Four models were then reconstructed. Model 1 contained 7 control points per polyline slice, model 2 had 20 control points, model 3 had 50 control points, and model 4 had 70 control points per slice. The more control points per polyline decreases the surface smoothness of the resulting AAA. Figure 2 shows the difference in polylines between the various degrees of smoothing. Non-linear stress analyses were then performed on each of the four models. Resulting wall stress distributions are shown in Figure 3, with stress results normalised to the peak stress experienced in the roughest model, namely model 4. Mesh independence was performed on all models. From these results, model 2 was deemed to be the optimum level of smoothing as unwanted surface detail is removed without causing unnecessary over-smoothing. Figure 4 illustrates the difference between the resulting rough and smooth models. These AAA surfaces were then exported in IGES file format for further work.

### Biomechanical material properties

#### AAA(SIMP)

Firstly, the AAA material was assumed to be homogenous and isotropic with linear elastic material properties. This model is referred to as *AAA*(*SIMP*)_*L*_. Although human arterial tissue acts like a non-linear material, at pressures above 80 mmHg (10.67 KPa) the aorta behaves more like a linearly elastic material [23,24,32,33]. The modulus of elasticity applied to the AAA wall was 4.66 MPa, as used in previous research [24,27]. Many researchers have modelled the aorta as linearly elastic in order to examine the influence of geometry and various other parameters on wall stress [2,4,23-25,34].

The same model was then analysed using non-linear elastic material properties, and is referred to as *AAA*(*SIMP*)_*NL*_. The AAA wall was modelled as a homogenous isotropic hyperelastic material using the finite strain constitutive model proposed by Raghavan and Vorp [3] in Eqn. 1. These material properties have been utilised for many stress analysis studies [5-7,13,20,26-28].

W = C_1_(I_B _- 3) + C_2_(I_B _- 3)^2^

Where, W is the strain energy and I_B _is the first invariant of the left Cauchy-Green tensor **B **(I_B _= tr **B**). The constants were set to the population mean values C_1 _= 174,000 Pa (0.174 MPa) and C_2 _= 1,881,000 Pa (1.881 MPa). Aortic tissue is also known to be nearly incompressible with a Poisson's ratio of approximately 0.49 [2,24,35].

#### AAA(MOD)

For this set of AAAs, the wall was again assigned both linear and non-linear material properties, referred to as *AAA*(*MOD*)_*L *_and *AAA*(*MOD*)_*NL*_, respectively. *AAA*(*MOD*)_*L *_was assigned an Young's modulus of 4.66 MPa, like earlier, and *AAA*(*MOD*)_*NL *_utilized the non-linear model proposed by Raghavan and Vorp [3].

#### AAA(COMP)

In the *AAA*(*COMP*) models, the ILT was included as a separate entity in the AAA model, and therefore required its own material property. Previous work has used the theory of linear elasticity to model the ILT [4,28], and so for the *AAA*(*COMP*)_*L *_the ILT was assigned a Young's modulus of 0.11 MPa, and a Poisson's ratio of 0.45. The AAA wall was also assumed to be linearly elastic with the same material properties as described earlier. For the *AAA*(*COMP*)_*NL *_model, the ILT was modelled as a hyperelastic material using the material constants derived from 50 ILT specimens from 14 patients performed by Wang et al. [36]. The AAA wall was again analysed using the non-linear model proposed by Raghavan and Vorp [3]. Therefore, the model consisted of two non-linear materials, both with realistic material properties.

### Mesh generation

#### AAA(SIMP)

Once the AAA was imported into ABAQUS v.6.6-2 for stress analysis, a mesh was generated on the AAA model. The elements chosen for the application were linear shell elements [2,3,23]. The surface was partitioned, by dividing the model into different regions in order to optimise the mesh creation. Each shell element was assigned a uniform thickness of 1.5 mm [2,26,29]. Mesh independence was performed in order to determine the optimum number of elements. In order to gain confidence in the mesh size of each AAA model, the number of elements was incrementally increased and the peak wall stress computed. The optimum mesh size was determined once the peak stress did not increase by more than 2%. This method of convergence has been used in previous studies [37,38]. It was noted that the locations of the peak stress did not significantly alter when examining each model for mesh convergence. Both the *AAA*(*SIMP*)_*L *_and the *AAA*(*SIMP*)_*NL *_models were meshed using shell elements, with element numbers for each model in the order of 17000 and 30000, respectively.

#### AAA(MOD)

In order to mesh the 3D wall of the *AAA*(*MOD*) models, 3D stress elements were required in order to mesh throughout the thickness of the AAA wall. The elements used for this part of the study were quadratic, 10-noded, tetrahedral elements. Mesh convergence was achieved using the same approach as described earlier. In this case, the *AAA*(*MOD*)_*L *_and the *AAA*(*MOD*)_*NL *_models required 29000 elements. The location of peak stress did not significantly alter in location during the convergence study.

#### AAA(COMP)

As with the AAA(MOD) models, the elements employed here were quadratic tetrahedral 3D stress elements, with 10-nodes per element. The AAA wall and ILT regions were meshed using a master-slave contact scenario, where the slave region (ILT) has a larger number of elements than the master region (AAA wall). Both the *AAA*(*COMP*)_*L *_and the *AAA*(*COMP*)_*NL *_models converged at 13000 elements using the same convergence criteria as described previously.

### Forces and boundary conditions

The blood pressure within the AAA acts on the AAA inner wall, therefore, the pressure was applied to the inner surface of all the virtual AAA models studied. A static peak pressure of 120 mmHg (16 KPa) was used. Ideally, patient-specific blood pressures would be applied to each particular AAA, by measuring blood pressure at the time of CT scan, but for this study the standard peak pressure of 120 mmHg was felt to be sufficient. The shear stress induced by blood flow was neglected in this study [7,24], although the effects of blood flow have been shown to reduce wall stress by 10% in uniformly thick walled ideal models and by up to 30% in variable wall thickness models [34]. In order to simulate the attachment of the AAA to the aorta at the renal junction and iliac bifurcation, the AAA model was fully constrained in the proximal and distal regions. Residual stresses that may exist within the aortic wall *in vivo *and tethering forces on the posterior surface caused by the lumbar arteries were neglected. Bulging is on the anterior surface due to the constraint the spinal cord places on posterior dilation.

## Results

**Figure 5:**
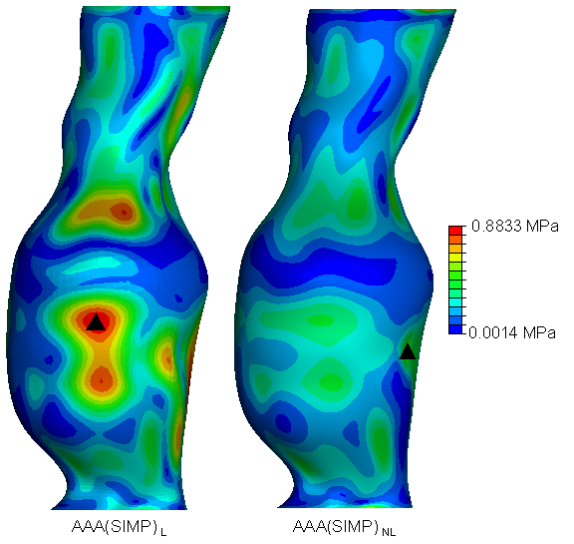
**von Mises wall stress distributions for both the *AAA*(*SIMP*)_*L *_and *AAA*(*SIMP*)_*NL *_models at an internal pressure of 120 mmHg**. Wall stress results are normalised to the peak stress found *AAA*(*SIMP*)_*L*_. The black mark indicates the region of peak wall stress. Models are shown in the anterior view.

**Figure 6:**
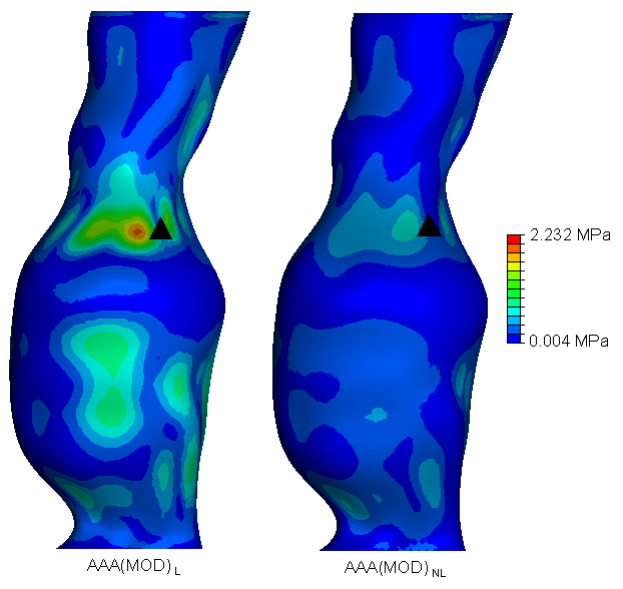
**von Mises wall stress distributions for the both the *AAA*(*MOD*)_*L *_and *AAA*(*MOD*)_*NL *_models at an internal pressure of 120 mmHg**. Wall stress results are normalised to the peak stress found *AAA*(*MOD*)_*L*_. The black mark indicates the region of peak wall stress. Models are shown in the anterior view.

**Figure 7:**
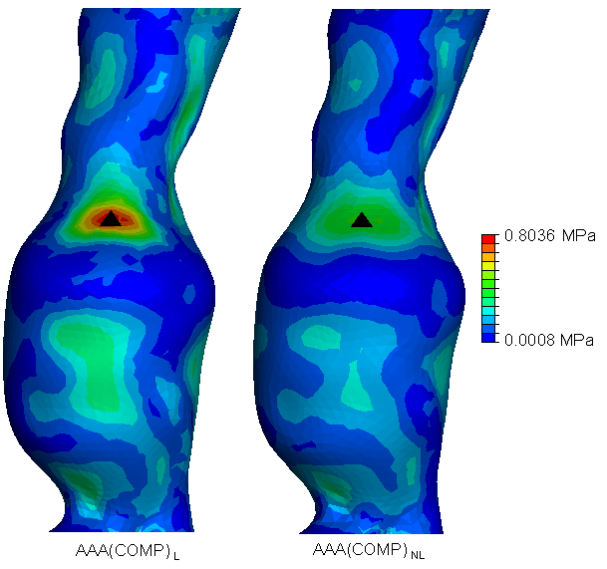
**von Mises wall stress distributions for both the *AAA*(*COMP*)_*L *_and *AAA*(*COMP*)_*NL *_models at an internal pressure of 120 mmHg**. Wall stress results are normalised to the peak stress found *AAA*(*COMP*)_*L*_. The black mark indicates the region of peak wall stress. Models are shown in the anterior view.

In order to easily observe and visualise the resulting wall stress of each AAA model, contours of the von Mises stress were plotted on the surface of each AAA model. The von Mises stress is a stress index especially suited for failure analysis, as stress is a tensor quantity with nine components, with the von Mises stress being a combination of these components [7]. The normalised computed wall stress results for the *AAA*(*SIMP*), *AAA*(*MOD*) and *AAA*(*COMP*) models can be seen in Figures 5, 6 and 7. In these figures, wall stress results were normalised by using the peak stress experienced in the linearly elastic model of each case. In each case, the linearly elastic model returned a higher peak stress than the corresponding non-linear model. The location of peak stress remained the same in all models except the *AAA*(*SIMP*) models, where the location of peak stress shifted from a centred anterior location to a left lateral location. For both linearly elastic and non-linearly elastic *AAA*(*MOD*) and *AAA*(*COMP*) models, the peak stress region was located on the inner surface of the AAA wall. When using 3D stress elements the stress is not interpolated through the wall thickness, as with shell elements, but rather at the individual integration points of the element. For the *AAA*(*COMP*) models the peak stress was located at regions between the intersection of the ILT region and the AAA wall.

In both the *AAA*(*SIMP*) models, the peak stress occurred at regions of relatively maximum diameter, rather than at inflection points along the surface of the AAA sac. For all *AAA*(*MOD*) and *AAA*(*COMP*) cases studied, the peak stress occurred at an inflection point on the inner surface of the AAA. An inflection point is defined as points on the AAA surface at which the local AAA wall shape changes from convex to concave [2]. Peak stress located at inflection points has been previously reported in idealised models, both numerically [2,19,34] and experimentally [18]. In general, wall stress reduces across the entire surface of the AAA when the material properties become non-linear. The recorded peak stresses for each case are also noticeably different. The peak stress results, along with location of peak stress and overall computational time can be seen in Table 1. Computational time here is rounded to the nearest minute. Peak stresses varied considerably depending on the modelling approach. By comparing the first approach of AAA reconstruction consisting of a single layer of shell elements, *AAA*(*SIMP*), the peak stress reduced from 0.8833 MPa to 0.5814 MPa when more realistic non-linear material properties were implemented. This drop in peak stress when modelled from a non-linear approach followed through to the second case also, that is, *AAA*(*MOD*). In this comparison of AAAs with 3D 1.5 mm thick uniform walls, the peak stress reduced from 2.232 MPa to 1.034 MPa. In the third case, *AAA*(*COMP*), again peak stress was reduced when realistic material properties were utilised. In this case the peak stress reduced from 0.8036 MPa to 0.4219 MPa. These large reductions in peak stress for each comparison can be attributed to the use of realistic material properties, as the geometry of the models are identical. The peak stress in the *AAA*(*SIMP*), *AAA*(*MOD*) and *AAA*(*COMP*) models reduced by 34%, 55% and 47%, respectively, when accounting for material non-linearity. This results in an average reduction in peak stress of 45% in all models.

**Table 1:** Comparison of peak wall stress, location of peak wall stress and FEA computational time for the *AAA*(*SIMP*), *AAA*(*MOD*) and the *AAA*(*COMP*) models.

	***Peak Wall Stress *(*MPa*)**	***Location of Peak Stress***	***CPU Time *(*min*)**
***AAA*(*SIMP*)_*L*_**	0.8833	Centre of anterior region	1
***AAA*(*SIMP*)_*NL*_**	0.5814	Left lateral region	9
***AAA*(*MOD*)_*L*_**	2.282	Inflection point on inner surface of anterior region	4
***AAA*(*MOD*)_*NL*_**	1.034	Inflection point on inner surface of anterior region	19
***AAA*(*COMP*)_*L*_**	0.8036	Inflection point on inner surface of anterior region	8
***AAA*(*COMP*)_*NL*_**	0.4291	Inflection point on inner surface of anterior region	82

**Figure 8:**
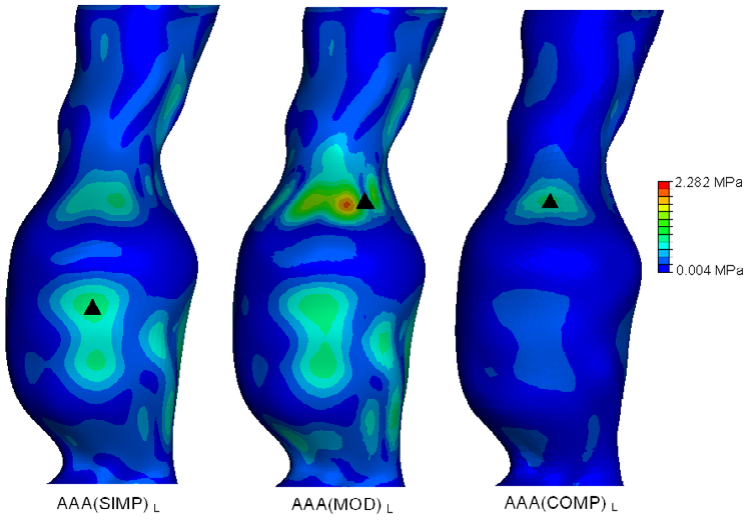
**Normalised wall stress results and locations of peak wall stress in all the linearly elastic models examined**. Wall stress results are normalised to the peak stress found in *AAA*(*MOD*)_*L*_. The figure shows the effect of modelling parameters and inclusion of the ILT on peak wall stress. The black mark indicates the region of peak wall stress. Location of peak stress for the *AAA*(*MOD*)_*L *_and *AAA*(*COMP*)_*L *_models are on the inner surface of the AAA wall. All models are shown in the anterior view.

**Figure 9:**
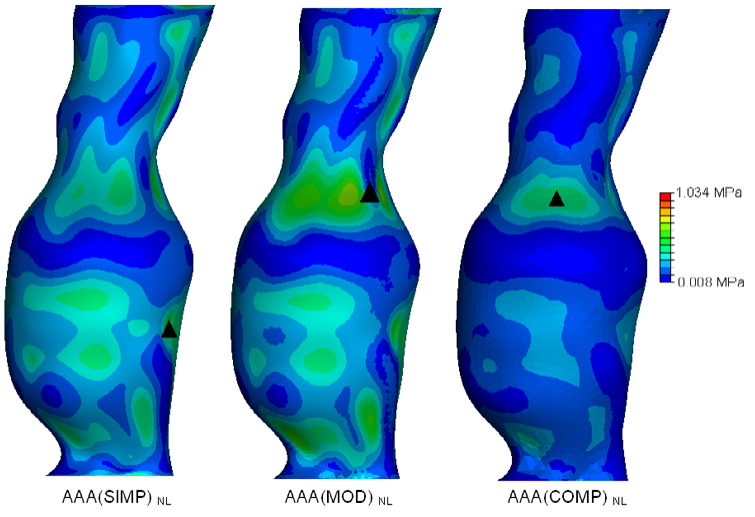
**Normalised wall stress results and locations of peak wall stress in all the non-linearly elastic models examined**. Wall stress results are normalised to the peak stress found *AAA*(*MOD*)_*NL*_. The figure shows the effect of modelling parameters and inclusion of the ILT on peak wall stress. The black mark indicates the region of peak wall stress. Location of peak stress for the *AAA*(*MOD*)_*NL *_and *AAA*(*COMP*)_*NL *_models are on the inner surface of the AAA wall. All models are shown in the anterior view.

The effect of geometrical modelling parameters can be seen in Figure 8 for the linearly elastic models, and in Figure 9 for the non-linearly elastic models. The location of peak stress is also indicated in these figures. The wall stresses were normalised using the peak wall stress experienced in the linear elastic study (Figure 8) and the peak stress obtained in the non-linear elastic study (Figure 9). By comparing the three models examined from a linearly elastic approach, the *AAA*(*MOD*)_*L *_returned the highest peak stress of 2.282 MPa. Both the *AAA*(*SIMP*)_*L *_and *AAA*(*COMP*)_*L *_returned similar peak stresses of 0.8833 MPa and 0.8036 MPa, respectively.

The "cushioning effect" of the ILT which has been previous documented by other researchers [13,17,35-40] can be observed by comparing the linear and non-linear cases of both the *AAA*(*MOD*) models and the *AAA*(*COMP*) models. The *AAA*(*MOD*)_*L *_and the *AAA*(*COMP*)_*L *_models are similar in material properties except for the inclusion of thrombus. There is a 65% reduction in stress when including the ILT in the linear case. This "cushioning effect" is also evident in the non-linear cases, *AAA*(*MOD*)_*NL *_and *AAA*(*COMP*)_*NL*_. Here, inclusion of ILT reduces peak stress by 59%.

## Discussion

In this study, one patient-specific AAA was selected for rigorous finite element analysis. The 3D reconstruction and stress analysis was performed using three different techniques, with each technique studied using both linearly elastic and non-linearly elastic material properties. This study is believed to be the first paper to contrast and compare established and widely used techniques of computing AAA wall stress distributions. The first technique is classed as *AAA*(*SIMP*), the second as *AAA*(*MOD*), and the third as *AAA*(*COMP*). Each model was then thoroughly analysed and compared to determine any differences in both peak wall stress and also wall stress distribution. The FEA technique used in this study has been validated both experimentally by Morris et al. [18] and numerically by Callanan et al. [19] in idealised AAA models in previous research by our group. Briefly, these previous studies used an idealised AAA model designed by our group using realistic dimensions from a large population study [41]. Aluminium moulds were then created using a CAD/CAM procedure, and experimental models manufactured using a technique described in previous research by both Morris et al. [18] and Doyle et al. [31]. The photoelastic method of determining stress distributions was then employed. A numerical model of the same AAA was then analysed using the finite element method, with good agreement between the two. Peak stresses were found to occur at regions of inflection on the surface of the AAA rather than at regions of maximum diameter. This result was also reported by other authors using both FEA [2] and fluid-structure interaction (FSI) [34]. 3D solid stress elements were used in the numerical aspect of this previous work as shell elements resulted in erroneous stress distributions.

### 3D reconstruction

The reconstruction process used here compared favourably to previous methods used by our group [30,31]. For the *AAA*(*SIMP*) and *AAA*(*MOD*) models, reconstruction involved the omission of the ILT, and so the AAA was modelled as a uniform walled vessel, consisting of one structure. For the *AAA*(*SIMP*) models, the single layer surfaces of these 3D reconstructions were then exported for stress analyses. The *AAA*(*MOD*) required the generation of a uniform artificial wall 1.5 mm thick in ProEngineer Wildfire 3.0, before being exported to the FEA solver. For the *AAA*(*COMP*), the ILT was incorporated into the vessel wall and so the complex AAA had an artificial uniform wall and ILT region of varying thickness. The full model was then exported for stress analyses. The iliac arteries were omitted from these models, as in previous studies [5]. The effect of smoothing on the 3D reconstruction was also examined. Four degrees of smoothing were studied in order to determine the effect smoothing plays on wall stress distributions. Normalised wall stress for the four models is shown in Figure 3. For model 1, the AAA was over-smoothed, and resulted in low wall stress. Smoothing was then reduced for model 2, and resulted in higher stress and less gradual changes in stress along the AAA surface. Smoothing was then reduced further for models 3 and 4, which both resulted in elevated stresses and also more surface detail. This surface detail results in rapid changes from regions of higher and lower stresses, showing jagged wall stress distributions. Also, if the stress distributions of models 3 and 4 were used in the study, they would indicate possible failure sites in abnormal regions of the AAA, as elevated stresses are shown in the healthy proximal neck. This observation is shown in Figure 3 by the black marks indicating these irregular locations of high stress. From this study the optimum level of smoothing was determined to be that of model 2, 20 control points per axial slice, and was used in all further reconstructions of this study.

### Biomechanical material properties

In each of the three geometrically different models, a linearly elastic model was examined. It has been argued in previous AAA wall stress studies [23,24] that the aorta behaves more like a linearly elastic material at pressures above 80 mmHg (10.67 KPa). Therefore, many researchers utilise the much simpler technique of linear elasticity in their studies [2,4,23-25]. Other researchers however, have reported that the use of simplified linearly elastic material properties or other inappropriate tissue constitutive models can lead to erroneous stress distributions [2,36-40]. For each of the non-linear models, the material properties of 69 AAA specimens form the basis of the finite strain constitutive model for AAA wall tissue proposed by Raghavan and Vorp [3] and has been utilised by many previous researchers [5-7,13,20,26-29]. These material properties are believed to resemble the behaviour of the actual AAA wall more closely than those of a linearly elastic material. When utilising the *AAA*(*COMP*) method, the ILT is an entirely different structure than that of the AAA wall, and therefore required its own material properties. The ILT was modelled as linearly elastic in the *AAA*(*COMP*)_*L *_using previously published data [4,28,35]. The non-linear material properties derived by Wang et al. [36] have been implemented to model the ILT region of the *AAA*(*COMP*)_*NL *_in a more realistic manner.

### Computational effort

The computational time for each stress analysis increases with the inclusion of non-linear material properties and complexity of the model, and so a compromise is usually desirable between computational time and accuracy. For this study the computational time for each analysis was recorded and compared. These results can be seen in Table 1. The differences in CPU times can be clearly seen, and highlight how the inclusion of non-linearity increases computational effort. Inclusion of the non-linear behaviour of the AAA wall resulted in a 9-fold increase in computational time for the *AAA*(*SIMP*) models, 5-fold increase for *AAA*(*MOD*) models, and a 10-fold increase for the *AAA*(*COMP*) models. Accounting for the "nonslip" contact between the AAA wall and the ILT region of the AAA(COMP) cases also increased the computational effort.

### Wall stress

The modelling techniques used in this study all return significantly different wall stress results. Peak wall stress was shown to reduce when taking into account the realistic non-linear behaviour of AAA tissue in all three modelling approaches. All wall stress results can be seen in Table 1. When modelled using non-linear material properties, wall stress reduced by 34% in the *AAA*(*SIMP*) case, 55% in the *AAA*(*MOD*) case, and by 47% in the *AAA*(*COMP*) case. These reductions in wall stress can be observed in Figures 5, 6, 7 for each of the three modelling techniques. From these figures it can be seen how the change in material properties does not significantly alter the stress distribution patterns on the surface of the AAA, instead it simply reduces the magnitude of these acting stresses.

The location of peak stress only differed when modelled using the *AAA*(*SIMP*) approach. This shift in peak stress location in the *AAA*(*SIMP*) models may indicate that the use of shell elements results in inaccurate stress distributions. Previous research undertaken by our group [19] also noted that shell elements can lead to erroneous stress distributions, in particular when using a linearly elastic material. In this previous numerical work [19], the ideal AAA examined showed peak stresses along the region of maximum diameter, rather than at the proximal and distal inflection points, as observed experimentally [18]. Callanan et al. [19] then used 3D stress elements to numerically analyse the ideal AAA model, and found that the stress distributions matched those found experimentally, with good correlation between the two sets of results. This background may indicate that the use of shell elements could possibly lead to inaccurate results in realistic AAA models also. For both the *AAA*(*MOD*) and *AAA*(*COMP*) models, 3D stress elements were used, and the location of peak stress did not significantly shift when the model accounted for non-linearity of the material. As the location of peak stress did not significantly alter in these models, it is believed that the use of 3D elements is more accurate than the use of shell elements in FE analyses of realistic AAAs. As mentioned earlier, this observation was reported in previous research by our group [19].

Figure 8 compares the wall stress results of the three linearly elastic models examined. Both the *AAA*(*MOD*) and *AAA*(*COMP*) models showed peak stress regions at inflection points on the inner surface at the proximal region of the AAA sac. These findings are consistent with previous experimental [18] and numerical work [19]. The *AAA*(*MOD*)_*L *_model returned the highest peak stress in all three cases. The peak stress in *AAA*(*MOD*)_*L *_is over 2.5 times the peak stress in both *AAA*(*SIMP*)_*L *_and *AAA*(*COMP*)_*L*_. Both the *AAA*(*SIMP*)_*L *_and *AAA*(*COMP*)_*L *_models returned similar peak stresses of 0.8833 MPa and 0.8036 MPa, respectively. In comparison, the wall stress results for the three non-linear cases are presented in Figure 9. Again, the *AAA*(*MOD*)_*NL *_model experienced the highest wall stress, with a peak stress 44% higher than the *AAA*(*SIMP*)_*NL*_and 59% higher than that experienced in the *AAA*(*COMP*)_*NL *_model. By including the ILT into the models, peak wall stress was reduced by 65% in the linear case and 59% in the non-linear case. This results in an average reduction of 62% in peak wall stress simply by including the ILT. The ILT absorbs the internal pressure induced by blood flow, and transfers the load to the AAA wall in a reduced amount. From the stress results found for all the various models analysed, the *AAA*(*COMP*)_*NL *_model is deemed to be the most accurate method of predicting wall stress in AAAs. This model incorporates the realistic non-linear behaviour of both the AAA wall and the ILT, and therefore produces the most accurate wall stress results.

### Significance of results

In order to determine the significance of the results reported here, other factors must be considered. It is known that AAA rupture occurs when the locally acting wall stress exceeds the locally acting wall strength. To make valid conclusions from the wall stress results presented here, wall stress was examined with respect to AAA wall strength. The AAA case examined in this study underwent routine CT scanning, thus detecting the aneurysm. Previous work has identified that AAA wall strength has an average failure stress of 0.942 MPa [16]. If the AAA was analysed for wall stress without inclusion of the ILT, such as cases *AAA*(*MOD*)_*L *_and *AAA*(*MOD*)_*NL*_, results would suggest that rupture has already occurred as local stress had exceeded local strength. By implementing the ILT into the model, peak stress was reduced to a level believed to more accurately represent that *in vivo*. The reported failure strength of 0.942 MPa [16] is higher than the predicted wall stress of 0.4291 MPa, indicating that the rupture potential of the AAA may be equalised. If wall stress results are to be incorporated into the clinical decision-making process, inclusion of non-linear material properties and the ILT may be important to the accuracy of the results.

### Limitations

The study presented here is not without limitations. Firstly, it is known that calcifications occur in almost all AAAs. These calcium deposits are believed to act as stress raisers within the wall and so incorporation into AAA stress studies may have significance [29]. Also, patient-specific blood pressures should be recorded at the time of CT scan. This would allow the accurate stress analysis of patient-specific AAAs, and as most patients exhibit elevated blood pressures, this could have an impact on peak wall stress. It is known that the AAA wall thickness is non-uniform [42], and therefore, incorporating a non-uniform wall into the modelling process may also alter stress distributions and peak stresses. The use of pulsatile pressures, possibly leading to fatigue testing, could also be incorporated into the modelling process. The relatively new computational field of fluid-structure interaction (FSI) could also lead to more accurate wall stress results [26-28,34]. Material properties could be improved. It is known that AAA tissue is non-homogenous and anisotropic, factors of which have been ignored for this study. The inclusion of more accurate material properties could lead to more precise wall stress distributions. This comparison also only accounts for one AAA model. Each individual AAA is unique, and a larger number of cases may give a better understanding of the role of certain modelling parameters and assumptions. Another limitation to this work, and also other AAA stress analyses, is that stress results are not intuitive to the clinician [5]. Use of the maximum diameter criterion is easy and clear to the surgeon, and therefore, wall stress results should be converted into a more clinician-friendly index. There is ongoing research on the rupture prediction of AAAs with the view to develop an easy to use tool that the clinician can implement into the decision-making process.

## Conclusion

This paper has examined the effect of modelling on the resulting wall stress distributions of a realistic AAA. Three different modelling techniques were implemented and each studied using both linear and non-linear material properties. It was found from the resulting stress distributions that inclusion of the ILT and implementing non-linear material properties may be important if accurate stress distributions are to be obtained. Peak stresses were shown to significantly vary depending on the modelling technique, with the most accurate model, namely *AAA*(*COMP*)_*NL*_, returning the lowest peak stress. Shell elements were shown to yield poor stress results, as there was a significant shift in peak stress location when non-linear material properties were implemented into the model. Also, in these *AAA*(*SIMP*) models the location of peak stress was shown to occur at regions of maximum diameter, which previous research [18,19] has shown not to be the case. Simplifying assumptions used in predicting wall stress with FEA should be limited.

The results reported here suggest that even patients with very modest levels of ILT, should be analysed using the more complex, time-consuming methods, such as including the ILT into FEA models, and the use of realistic, non-linear material properties. The effect of omitting these important parameters may lead to erroneous peak stress results, and ultimately to incorrect surgical decision-making. The principal idea behind reconstruction and stress analysis of AAAs is to determine the wall stress distributions of the AAA, and the potential of rupture. As rupture will occur when the local wall stress exceeds the local wall strength, and AAA have been shown to have variations in local wall strength [42], the need for improved prediction of peak wall stress location may also have clinical importance.

To conclude, the modelling technique employed in patient-specific FEA of AAAs, may be a valuable additional tool for clinical use.

## Declaration of competing interests

The author(s) declare that they have no competing interests.

## Authors' contributions

BJD designed the study, reconstructed the AAA models, conducted the wall stress simulations, analysed the results and prepared the manuscript. AC contributed to the wall stress studies and analysis of the results. TMM supervised the study, revised and gave the final approval of the manuscript. All authors read and approved the final manuscript.
